# Higher Pericyte Content and Secretory Activity of Microfragmented Human Adipose Tissue Compared to Enzymatically Derived Stromal Vascular Fraction

**DOI:** 10.1002/sctm.18-0051

**Published:** 2018-09-26

**Authors:** Bianca Vezzani, Isaac Shaw, Hanna Lesme, Li Yong, Nusrat Khan, Carlo Tremolada, Bruno Péault

**Affiliations:** ^1^ MRC Center for Regenerative Medicine University of Edinburgh Edinburgh United Kingdom; ^2^ Image Institute Milan Italy; ^3^ Orthopaedic Hospital Research Center and Broad Stem Cell Research Center David Geffen School of Medicine, University of California Los Angeles California USA

**Keywords:** Adipose stem cell, Mesenchymal stem cell, Cytokine, Pericyte, Secretome

## Abstract

Autologous adipose tissue is used for tissue repletion and/or regeneration as an intact lipoaspirate or as enzymatically derived stromal vascular fraction (SVF), which may be first cultured into mesenchymal stem cells (MSCs). Alternatively, transplant of autologous adipose tissue mechanically fragmented into submillimeter clusters has recently showed remarkable efficacy in diverse therapeutic indications. To document the biologic basis of the regenerative potential of microfragmented adipose tissue, we first analyzed the distribution of perivascular presumptive MSCs in adipose tissue processed with the Lipogems technology, observing a significant enrichment in pericytes, at the expense of adventitial cells, as compared to isogenic enzymatically processed lipoaspirates. The importance of MSCs as trophic and immunomodulatory cells, due to the secretion of specific factors, has been described. Therefore, we investigated protein secretion by cultured adipose tissue clusters or enzymatically derived SVF using secretome arrays. In culture, microfragmented adipose tissue releases many more growth factors and cytokines involved in tissue repair and regeneration, noticeably via angiogenesis, compared to isogenic SVF. Therefore, we suggest that the efficient tissue repair/regeneration observed after transplantation of microfragmented adipose tissue is due to the secretory ability of the intact perivascular niche. Stem Cells Translational Medicine *2018;7:876–886*

**Significance Statement**

The fat collected during a liposuction contains tissue‐repairing cells used diversely to erase wrinkles, reconstruct breasts, or treat arthritic joints. These cells, dissociated after enzymatic digestion of the fat, secrete diverse regenerative molecules playing important roles in tissue repair. Results of this study show that keeping the micro‐architecture of the fat intact, by physical fragmentation into sub‐millimetric units in the absence of enzyme treatment, guarantees optimal maintenance of regenerative cells and dramatically improves factor secretion thereby. These results likely explain the observed therapeutic superiority of micro‐fragmented adipose tissue and suggest it should be preferred to routinely used, enzymatically produced single cell suspensions.

## Introduction

Pioneered at the end of the nineteenth century for face and breast reconstruction, adipose tissue (AT) grafting later met with scepticism and mostly fell out of favor until new successful attempts reinvigorated this surgical approach in the 1980s 1, 2. First used intact or minimally processed as a filler for tissue contouring and augmentation, AT became a genuine cell therapy product following the demonstration that it contains multilineage progenitor cells that behave as mesenchymal stem cells (MSCs) 3. Routine processing of lipoaspirates (LPA) for therapeutic purposes consists of enzymatic dissociation into single‐cell suspensions, subsequent elimination of adipocytes by centrifugation, and collection of the remaining stromal vascular fraction (SVF), which can be used immediately or following in vitro cell expansion. The latter protocol involving long‐term SVF culture allows isolation from AT of bona fide MSCs, also named adipose‐derived stem cells, which are morphologically, antigenically, and developmentally similar to their bone marrow‐derived counterparts (reviewed in 4), and can equally well control graft‐versus‐host reaction via their immunosuppressive properties 5. MSCs are isolated through enzymatic digestion and are defined by specific criteria: plastic adhesion once cultured; expression of markers as CD73, CD90, CD105, and ability to differentiate in vitro into osteocytes, adipocytes and chondrocytes under appropriate culture stimuli 6. Bone marrow and AT are no exceptions because MSCs can be derived from multiple other organs after enzymatic dissociation and culture selection, such as dental pulp 7, lung 8, skeletal muscle 9, and endometrium 10, to cite but a few.

Vascularized tissues in general have the capacity to give rise to MSCs in culture, and fittingly peri‐endothelial cells, namely pericytes that enclose capillaries and microvessels 11, 12, 13, 14, 15, 16, 17, and adventitial stromal cells that surround larger arteries and veins 18, 19, 20, 21, 22, were identified as in vivo progenitors of cultured MSCs. This view is supported by the fact that both pericytes and adventitial cells express MSC markers in vivo and are endowed with mesodermal differentiation abilities upon culture 13, 23, 24, 25, 26, 27. Moreover, similarity in gene expression profile between MSCs and pericytes has recently been reported 28. Analysis of transcriptomes in single perivascular cells purified from human AT confirmed this progenitor status, as well as revealing a hierarchical organization whereby adventitial stromal cells are developmentally more primitive than pericytes 29.

It is, however, important to note that although resident pericytes can replenish different cell lineages in vivo, such as adipocytes 30, dental pulp 31, 32, satellite cells and myofibers 33, follicular dendritic cells 34, myofibroblasts 35, 36, 37, 38, and yield MSCs in culture, their behavior as bona fide MSCs (i.e., immunosuppressive and secretory cells endowed with osteo‐, chondro‐, adipo‐, and myogenic developmental potentials) in situ has not been documented. In addition, distinguishing properties can be observed in isolated MSCs depending on the tissue of origin and the isolation technique used 26, 27, thus suggesting the presence of a heterogeneous population of progenitor cells in most adult organs 39, 40, 41, 42. Therefore, according to current knowledge, although purified pericytes and adventitial cells have been proven to yield MSCs in culture, whether they are endowed with the same exact potential in situ is unknown.

In summary, AT is used therapeutically for the treatment of different conditions, either as an intact tissue or enzymatically derived SVF, used either immediately or cultured into MSCs. Alternatively, undissociated AT transplants have been supplemented with MSC injections to gain higher therapeutic efficiency (reviewed in 43).

A recent innovation has been the use of mechanically fragmented AT, thus avoiding any enzymatic processing, for the treatment of diverse medical conditions. One of the most commonly used procedures to mechanically dissociate AT is Lipogems technology. Lipogems is a device used to process manual LPA into microfragmented adipose tissue (MAT) clusters through a mild mechanical size reduction using a sequence of sieves and steel marbles. It is a full immersion closed system that can be used directly in the operating room, reducing contamination risk that results from tissue exposure and/or extended processing methods. The generated AT clusters are a few hundred micrometers in diameter and free from blood and free lipids. Autologous transplantation of such MAT has been used with success in multiple indications, spanning cosmetics, orthopaedics, proctology, and gynaecology 44, 45, 46, 47, 48, 49, 50, 51, 52, 53, 54.

To further investigate the regenerative potential of MAT compared to that of enzymatically derived SVF, we characterized the perivascular cell distribution and in vitro protein secretion of Lipogems processed human AT versus isogenic collagenase digested LPA. We show how mechanical fragmentation of LPA modifies the resulting perivascular cell content of the tissue, and additionally that enzymatic dissociation negatively influences growth factor and cytokine secretion in vitro.

## Materials and Methods

### Human Tissues

AT was collected with prior written informed consent from healthy female patients (26–71 years old) undergoing cosmetic liposuction or abdominoplasty. Ethical approval for the use of human tissues in research was obtained from the South East Scotland Research Ethics Committee (reference: 10/S1103/45).

### Subcutaneous Abdominal Fat Collection

Subcutaneous AT from abdominoplasty samples was injected with 50 to 100 ml of 0.9% NaCl solution, warmed at 37°C, using a disposable tissue infiltration cannula (17Gx185 mm‐VG 17/18). LPA were obtained either manually using a 10‐cc luer lock syringe connected to a disposable liposuction cannula (LGI 13Gx185 mm ‐ AR 13/18) or using a standard vacuum pump‐assisted liposuction technique. All instruments used in the manual lipoaspiration procedure were provided in the Lipogems Surgical Kit (Lipogems, Milan, Italy).

### Microfragmentation of Adipose Tissue

A total of 60 ml of manual lipoaspirate was processed each time with the Lipogems 60 device following manufacturer's instructions. Briefly, the system was connected to a 0.9% NaCl solution supply until the cylinder was completely filled and no air was present in the system. First, 30 ml of the manual lipoaspirate were pushed into the cylinder through the blue size reduction filter inlet. The cylinder was shaken for 1 minute to emulsify oil. During the whole process, blood components and emulsified oil residues were removed by the flow of saline. When the solution inside the cylinder appeared clear, floating MAT was expelled from the cylinder through the gray size reduction filter outlet into 10 ml syringes connected to the device. This procedure was repeated, until 60 ml of lipoaspirate were fully processed, yielding from 20 to 30 ml of MAT.

### Cell Isolation

Fresh AT specimens (LPA and MAT) were dissociated enzymatically to obtain SVF. Briefly, samples were digested with type‐II collagenase (1 mg/ml collagenase in DMEM, both from Gibco, Thermofisher Scientific, Waltham, MA) for 45 minutes at 37°C in a shaking water bath. Samples were then washed with 2% FCS/PBS (Sigma Aldrich, St Louis, MO) and filtered sequentially through 100‐ and 70‐μm cell strainers (BD Falcon, Corning, NY). After centrifugation, pellets were resuspended in erythrocyte lysis buffer (155 mM NH_4_Cl, 170 mM Tris, pH 7.65, all from Sigma‐Aldrich) for 15 minutes at room temperature. Cells were washed again with 2% FCS/PBS and filtered through 40‐μm cell strainers (BD Falcon) to obtain single cell suspensions. Viable cells were counted following trypan blue staining (BioRad, Hercules, CA) on a haemocytometer.

### Flow Cytometry Analysis

The SVF was stained with the following antibodies: CD31‐V450 (1:400) or CD144‐PerCP Cy5.5 (1:100), CD34‐PE (1:100), CD45‐V450 (1:400) or CD45‐APC Cy7 (1:100), and CD146‐BV711 (1:100) (all from BD Biosciences, San Jose, CA). Cells were stained for 30 minutes at 4°C in the dark, followed by washing with 2% FCS/PBS. Analysis was performed on a BD LSR Fortessa 5‐laser flow cytometer (BD Biosciences) using Diva software (v.6.0, BD Biosciences). Single stained beads were used as compensation controls. Data were analyzed using FlowJo (v.10.0, FlowJo, Ashland, OR). Forward scatter area (FSC‐A) versus side scatter area (SSC‐A) gate was used to identify cells, followed by FSC‐A versus forward scatter height (FSC‐H) to select single cells. Viable cells were gated as negative for 4′,6‐diamidino‐2‐phenylindole (DAPI, Life Technologies, Carlsbad, CA) staining. Hematopoietic and endothelial cells were excluded by gating on CD31 and CD45 negative cells. Perivascular cells were identified as pericytes (CD146^+^ CD34^−^) or adventitial cells (CD146^−^ CD34^+^).

### Fluorescent Immunohistochemistry

AT specimens (unprocessed AT, LPA and MAT) were fixed in 4% buffered paraformaldehyde (PFA) at 4°C overnight. Samples were immersed for 24 hours in 15% sucrose in PBS (w/w), then embedded in 15% sucrose and 7% gelatin in PBS. After 4 hours at 37°C, samples were transferred to 4°C. After 24 hours, samples were frozen on dry ice. Embedded samples were stored at −80°C and cryosectioned at 8–10 μm thickness. Sections were fixed in 4% PFA prior to staining. Nonspecific antibody binding was blocked with 10% goat serum in PBS (Sigma‐Aldrich) for 1 hour at room temperature. The following uncoupled primary antibodies were used: mouse anti‐human‐NG2 (1:100; ref. 554275, BD Biosciences), rabbit anti‐human‐PDGFRβ (1:100; ref. 32570, Abcam, Cambridge, UK). All primary antibodies were diluted in antibody diluent (Life Technologies, https://www.google.co.in/search?q=Carlsbad+California&stick=H4sIAAAAAAAAAOPgE-LSz9U3MKmqSInPVeIAsYtMyvO0tLKTrfTzi9IT8zKrEksy8_NQOFYZqYkphaWJRSWpRcUA_pIQXEQAAAA&sa=X&ved=2ahUKEwifisTDzsvcAhWYXysKHTwRBU8QmxMoATARegQIDRAc) and incubated at 4°C overnight. After washing with PBS, sections were incubated for 1 hour at room temperature with species‐specific secondary antibodies diluted 1:300. The following fluorochrome‐conjugated secondary antibodies were used: anti‐mouse‐Alexa 555 IgG, anti‐rabbit‐Alexa 647 IgG, and streptavidin conjugated 488 (all from Life Technologies). Directly biotinylated *Ulex europaeus* lectin (UEA‐1) was used as an endothelial cell marker for long‐term cultured cells (1:200; Vector‐B1065, Vector Laboratories, Burlingame, CA). Nuclei were stained with DAPI (Life Technologies) for 10 minutes at room temperature. Slides were mounted using Fluoramount G (SouthernBiotech, Birmingham, AL) and images were acquired using a fluorescence microscope (Zeiss Observer, Zeiss, Oberkochen, Germany; Olympus BX61, Olympus, Tokyo, Japan). Images were processed using Fiji software 55 or ZEN Blue lite version (Zeiss).

### Tissue Culture and Medium Collection

SVF cells derived from MAT or LPA were plated at a density of 6,000 cells/cm^2^ and cultured in basal medium, consisting of DMEM Glutamax (Gibco) supplemented with 100 μg/ml streptomycin (Sigma‐Aldrich), 100 U/ml penicillin (Sigma‐Aldrich) and 20% heat‐inactivated foetal calf serum (Sigma‐Aldrich). 200 mg (corresponding to 200 μl of MAL) were plated in each well of a six‐well plate and cultured in basal medium. After 8 days in culture under standard conditions (37°C, 5% CO_2_) culture media from SVF and MAT were collected and stored at −20°C.

### Secretome Arrays

Secretomes were analyzed using the Proteome Profiler Human XL Cytokine Array kit (ARY022b) and Human Angiogenesis Array kit (ARY007), following manufacturer's instructions (R&D Systems, Minneapolis, MN). Conditioned media collected from cultured SVF and MAT were centrifuged at 500*g* for 5 minutes at room temperature to remove debris, filtered through a 70‐μm cell strainer to get rid of adipocytes/small residues of MAT, and incubated with both arrays. The signal was detected using the LiCOR Odyssey Fc apparatus (LICOR, Lincoln, NE), exposing array membranes for 10 minutes. Positive signals on the membranes were quantified using Image Studio Lite Software (LICOR). The average signal (pixel density) of the duplicate spots corresponding to each protein was normalized on the average signal of paired spots on the negative control. Normalized signals of each protein were then used for comparative analysis.

### Statistics

Statistical analysis was performed by using the Student's *t* test using Microsoft Excel or GraphPad Prism5 software. Results are presented as means ± SEM. A *p* value of less than .05 was considered statistically significant.

## Results

### The Perivascular Niche Is Preserved in Microfragmented Fat

**Figure 1:**
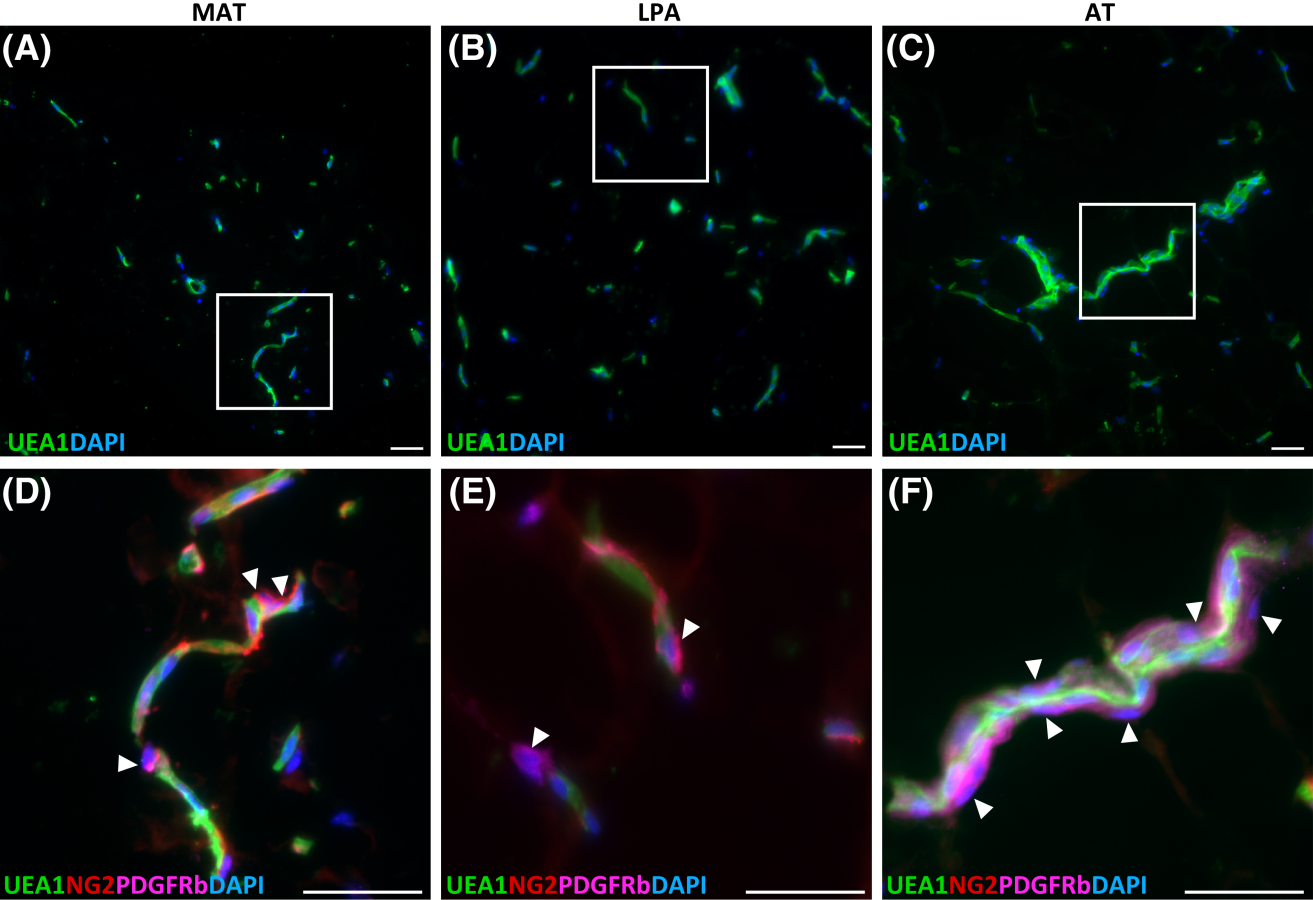
Vasculature in unprocessed and microfragmented adipose tissue. **(A, B, C):** Endothelial cells are stained with UEA‐1. From left to right: microfragmented adipose tissue (MAT), lipoaspirate (LPA), adipose tissue (AT). Larger vessels were observed only in LPA and AT. **(D, E, F):** Boxed areas in A, B, C are showed enlarged in D, E, F respectively. Arrowheads indicate pericytes, which have been stained using antibodies against PDGFRβ and NG2. Scale bar: 50 μm.

Detection of the endothelial cell marker *Ulex europaeus* agglutinin 1 (UEA‐1) receptor on sections of MAT, LPA, and AT illustrated the vascular network present in AT, with microvessels located between adipocytes. Larger vessels were observed principally in the unprocessed AT and LPA, while MAT was mainly characterized by the presence of small, capillary‐like vessels (Fig. 1A–1C).

Staining for pericyte markers revealed that after AT mechanical fragmentation, pericytes expressing NG2 or PDGFRβ are normally distributed, still ensheathing endothelial cells in microvessels (Fig. 1D). The same was observed in AT and LPA specimens, suggesting that microfragmentation is not affecting the perivascular cell compartment in microvessels (Fig. 1E, 1F).

### MAT Is Enriched in Pericytes Compared to Lipoaspirate

**Figure 2:**
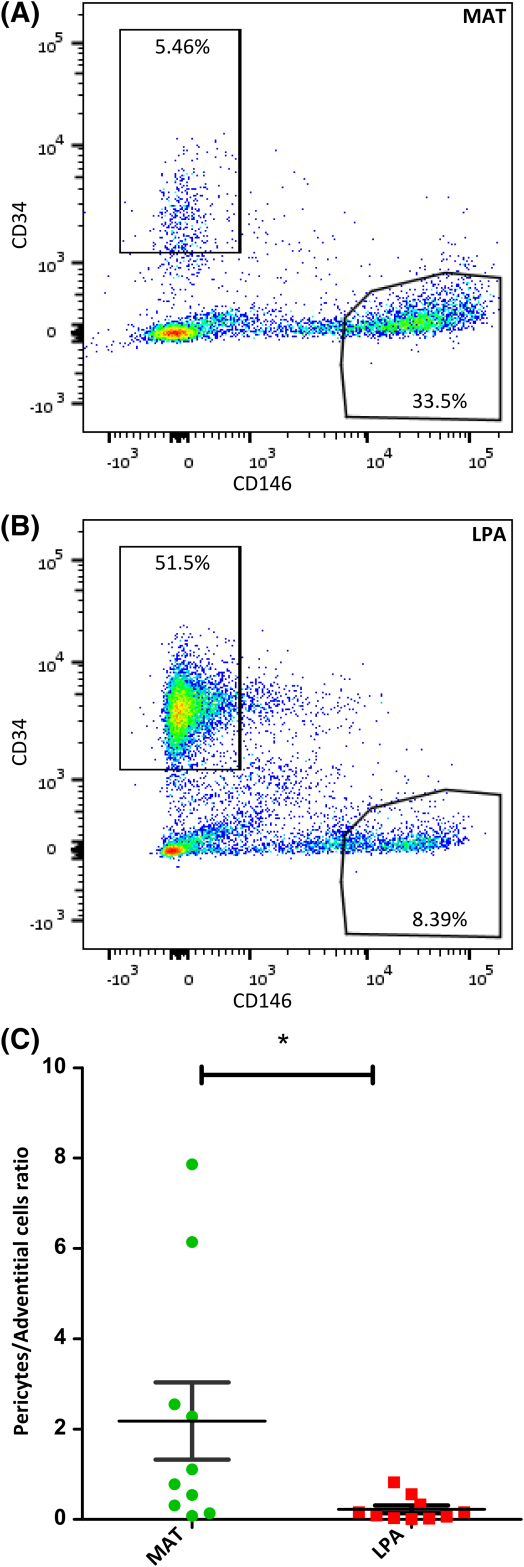
Respective abundances of perivascular adventitial cells and pericytes in MAT and LPA. **(A, B):** Dotplots showing adventitial cells (CD34^+^CD146^−^) and pericytes (CD146^+^CD34^−^) populations in MAT an LPA from the same donor. **(C):** Quantitative distribution of pericytes and adventitial cells in LPA and MAT, *n* = 10, *p < .05.

AT samples (MAT and LPA) were digested using collagenase and analyzed by flow cytometry. The average yield of nucleated cells in the SVF was 27 × 10^3^ ± 15 × 10^3^ cells per milliliter of MAT (*n* = 7) and 69 × 10^3^ ± 56 × 10^3^ cells per milliliter of LPA (*n* = 7). Viable cells were selected excluding debris, dead cells, and doublets. Endothelial cells and leukocytes were excluded from the analysis using CD31 and CD45, respectively. Pericytes were identified as CD146^+^CD34^−^, and adventitial cells as CD34^+^CD146^−^ cells 56. MAT was observed to be enriched in pericytes compared to LPA. On an isogenic specimen analysis, pericytes and adventitial cells in LPA account for 8.39% and 51.5% of the cells, respectively, in agreement with previously observed values 13, 18, 56 (Fig. 2B). In the MAT counterpart, pericytes and adventitial cells amounted to 33.5% and 5.46%, respectively (Fig. 2A). This difference between LPA and MAT, regarding pericyte and adventitial cell numbers, was observed to be significant (*p* < .05, *n* = 10; Fig. 2C).

### Microfragmented Fat Secretes in Culture a Higher Number and Higher Amounts of Proteins than Isogenic SVF

**Figure 3:**
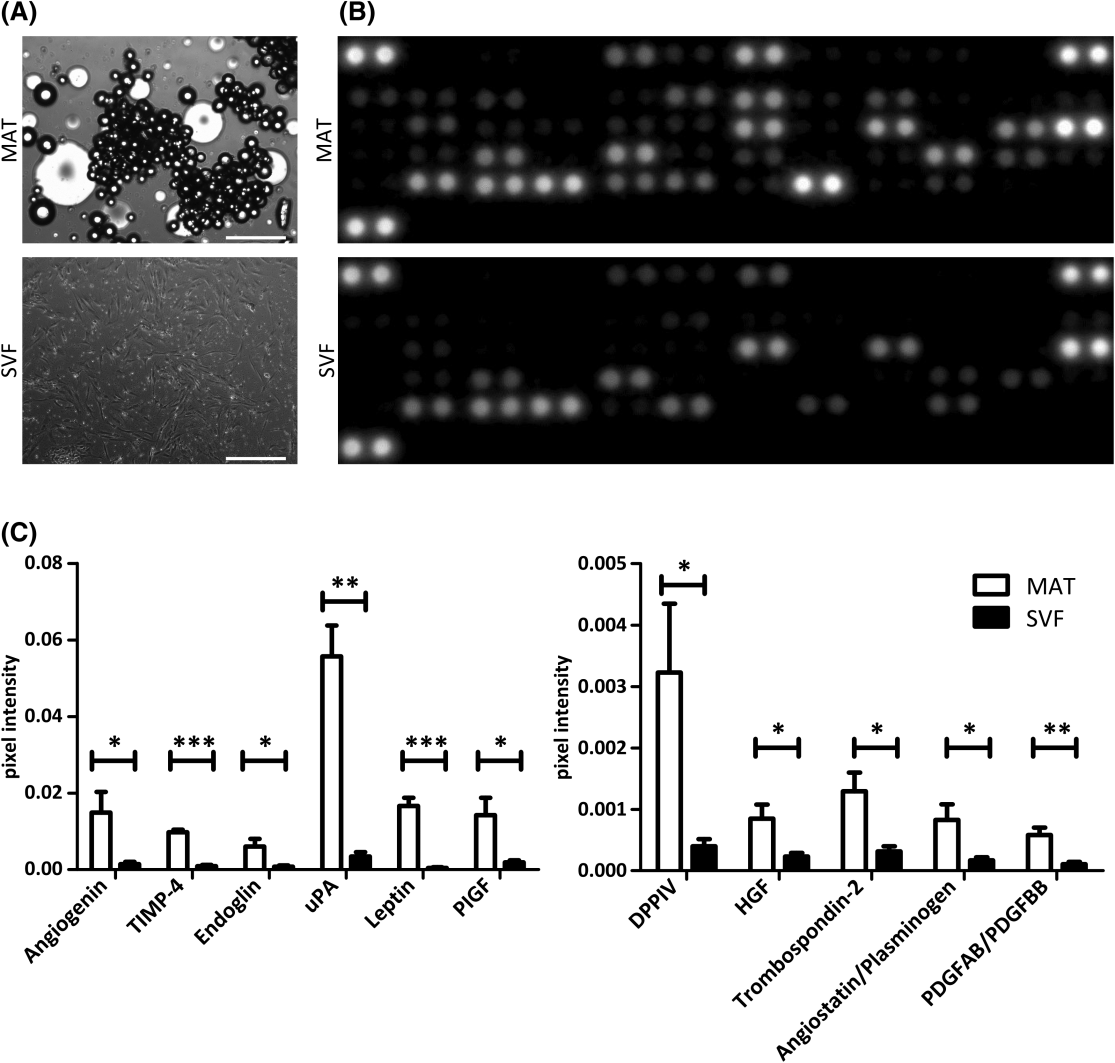
Angiogenic protein secretion by cultured MAT and isogenic SVF. **(A):** MAT and SVF cultured in basal medium. Scale bar: 500 μm. **(B):** Angiogenesis proteomic array showing secreted proteins from MAT and SVF after 8 days in culture. Capture antibodies are spotted in duplicate, each dot doublet represents a detected protein. **(C):** Secretion level of different angiogenic proteins measured as the average of the pixel intensity of the doublets and normalized to the negative control. Statistical analysis was performed on pooled secretion values detected in four separate donors. *p < .05; **p < .01; ***p < .001.

**Figure 4:**
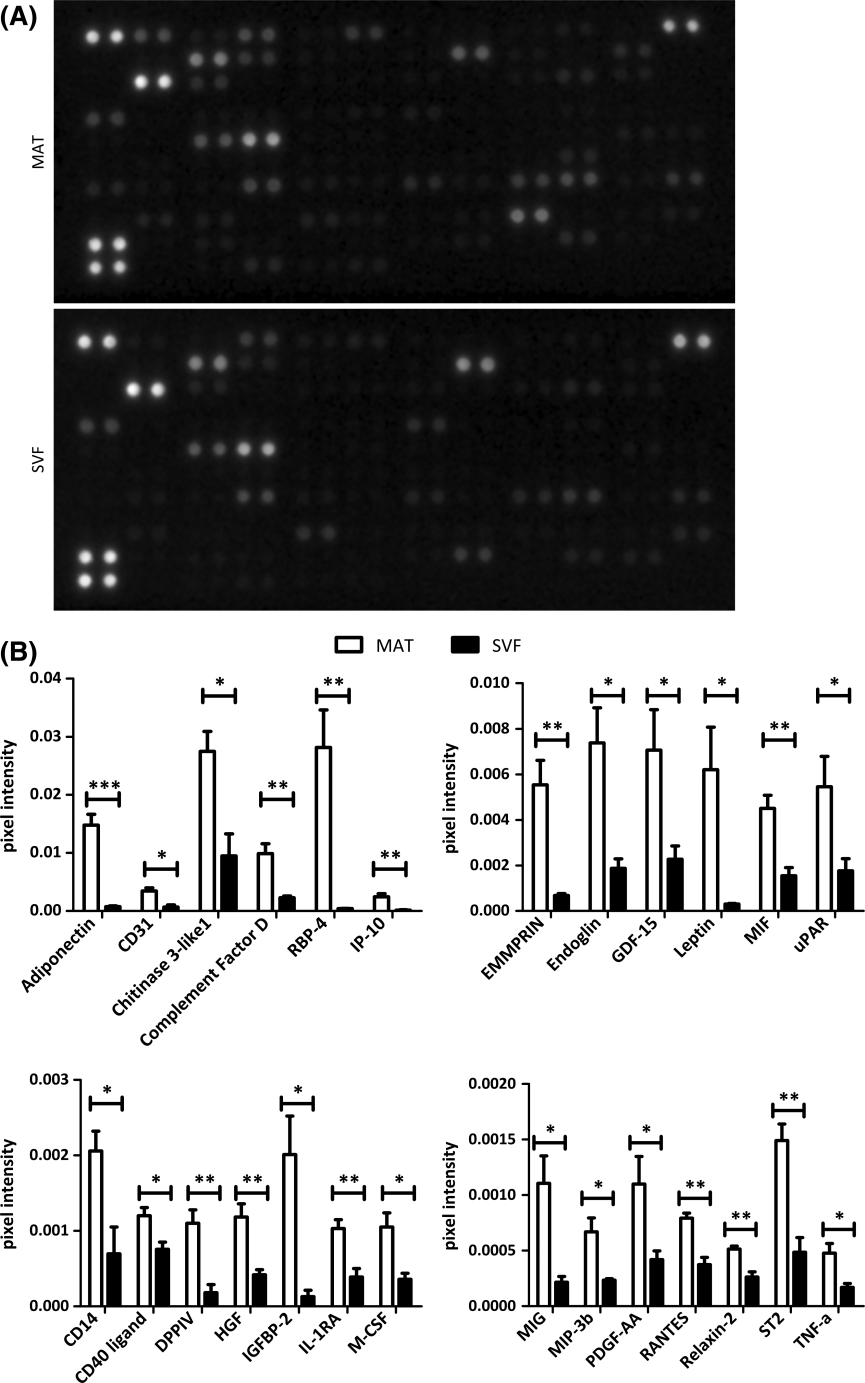
Cytokine secretion by cultured MAT and isogenic SVF. **(A):** Cytokine proteomic array showing secreted proteins from MAT and SVF after 8 days in culture. Capture antibodies are spotted in duplicate, each dot doublet represent a detected protein. **(B):** Secretion level of different cytokines measured as the average of the pixel intensity of the doublets and normalized on the negative control. Statistical analysis was performed on pooled secretion values detected in four separate donors. *p < .05; **p < .01; ***p < .001.

**Figure 5:**
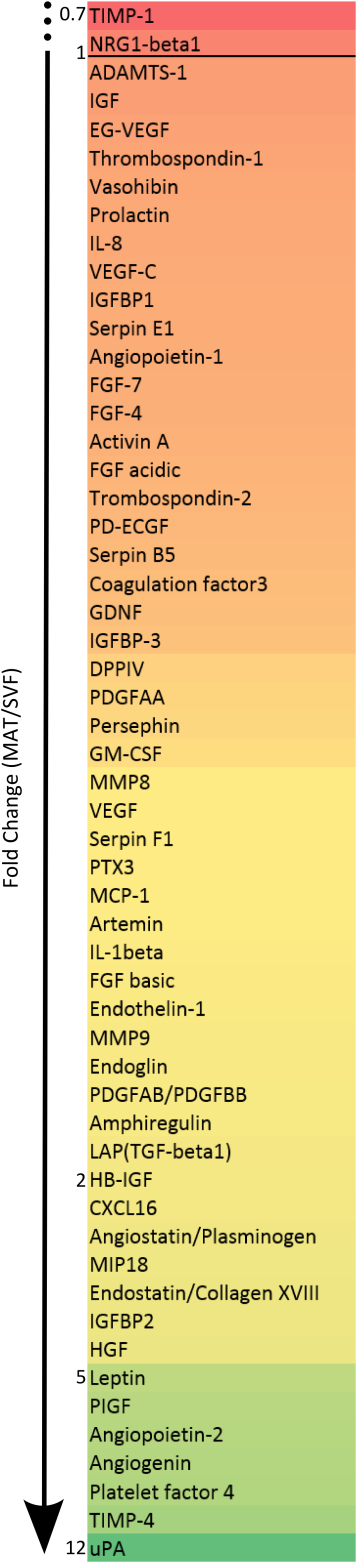
Angiogenic protein secretion levels of MAT compared to SVF. Secretion levels of angiogenic factors detected using the angiogenesis proteomic array, expressed as fold change between MAT and SVF values. The values represent the normalized pixel intensity detected in four separate donors. The numbers at the side of the list indicate fold changes.

**Figure 6:**
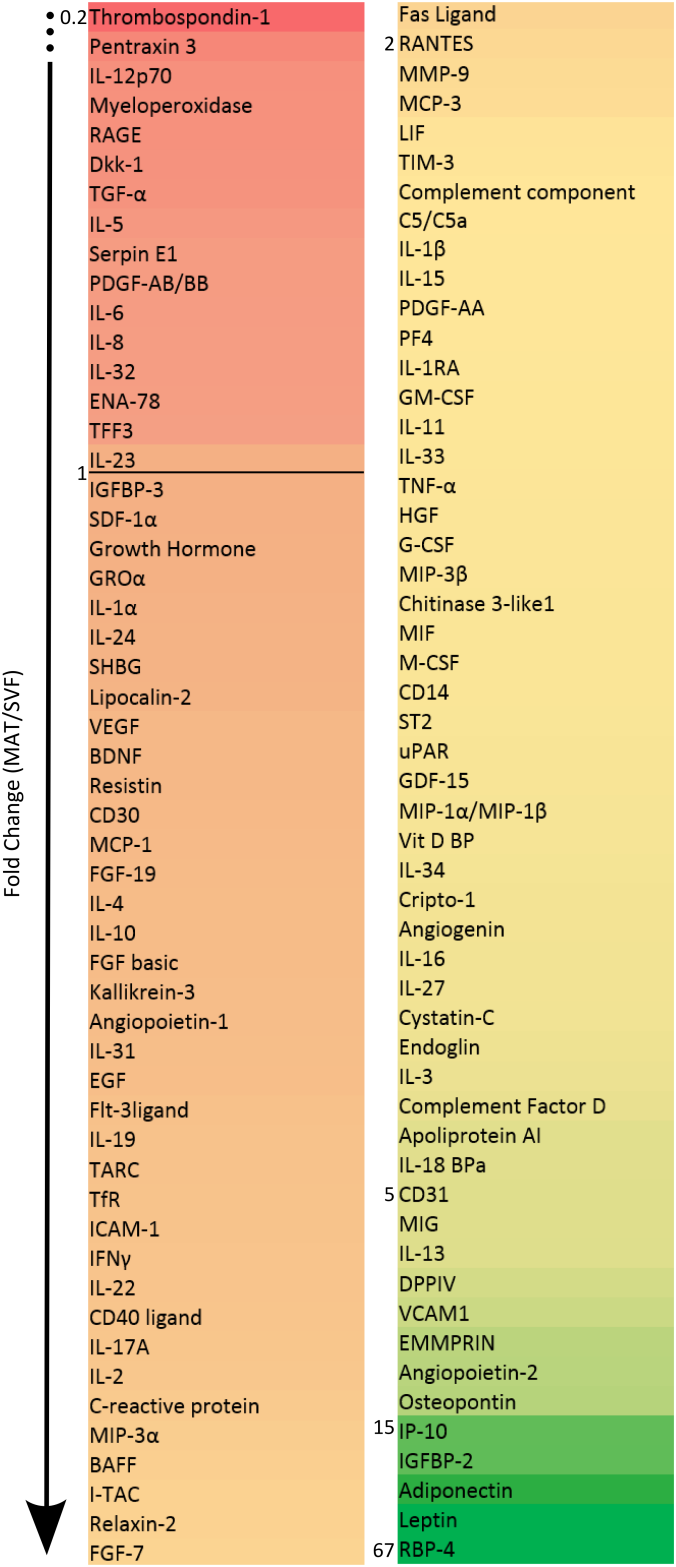
Cytokine secretion levels of MAT compared to SVF. Secretion levels of cytokines detected using the cytokine proteomic array, expressed as fold change between MAT and SVF values. The values represent the normalized pixel intensity detected in four separate donors. The numbers at the side of the list indicate fold changes.

**Figure 7:**
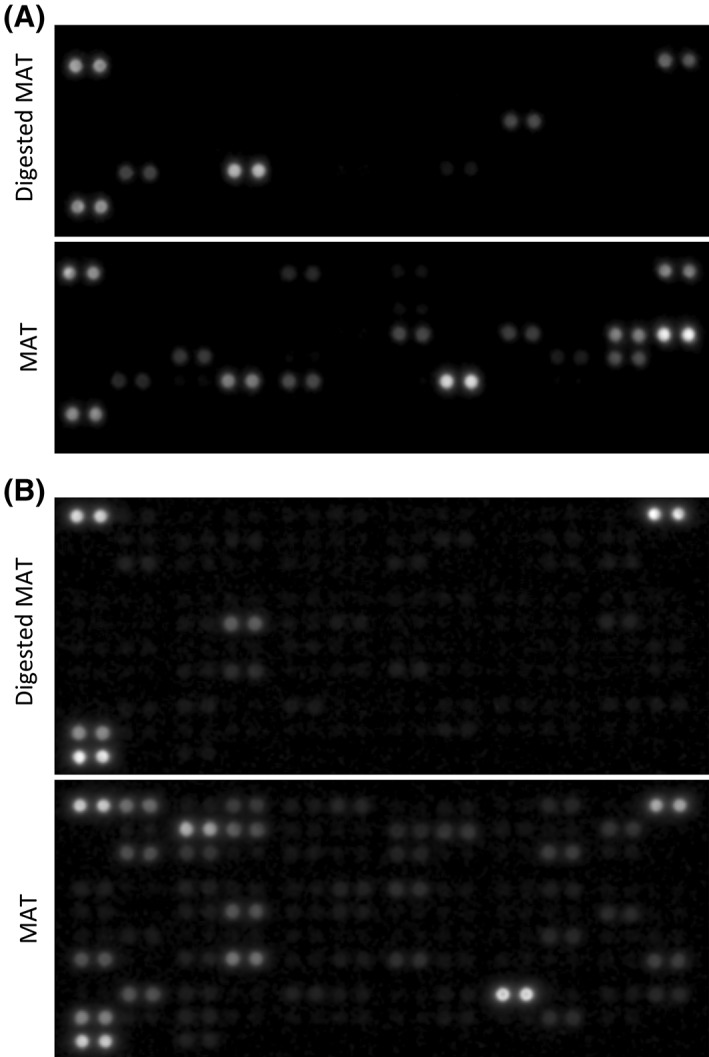
Protein secretion of enzymatically digested MAT and undigested MAT. **(A):** Angiogenesis proteomic array and **(B)** cytokine proteomic array showing secreted proteins from enzymatically digested MAT and undigested MAT after 8 days in culture in basal medium. Capture antibodies are spotted in duplicate, each dot doublet represent a detected protein.

MSCs are known to secrete growth factors and cytokines, either free or via microvesicle cargoes, involved in tissue repair and regeneration 57, 58. Assuming that native perivascular cells, the in vivo progenitors of MSCs, present in AT are responsible for its regenerative potential, we aimed to compare the secretome of MAT to that of enzymatically derived SVF. Isogenic MAT and SVF isolated from four different donors were cultured for 8 days in basal medium (Fig. 3A). Conditioned media were then analyzed, using proteome profiler commercial assays, for the presence of a range of cytokines and growth factors. Four independent experiments revealed that MAT secretes a greater number of cytokines and angiogenic growth factors than SVF (Figs. 3B and 4A). Moreover, comparative analysis on data derived from independent experiments conducted on four different biological samples revealed that most cytokines and angiogenic factors secreted by both MAT and SVF were more abundant in the supernatant of the former (Figs. 5 and 6). These results suggest that collagenase digestion reduces the secretory activity of AT stromal vascular cells, both qualitatively and quantitatively. To directly test this hypothesis, MAT was digested with collagenase, and the derived SVF was placed in culture. Both intact and enzymatically dissociated MAT were cultured in parallel for 8 days in basal medium and the resulting conditioned media were analyzed as described above. Enzymatic treatment of microfragmented fat dramatically reduced secretory activity, which became comparable to that observed from conventional SVF (Figs. 3B, 4A, 7A and 7B).

On detailed analysis of secreted angiogenic growth factors, we found higher secretion by MAT of angiogenin, angiostatin/plasminogen, DPPIV, endoglin, hepatocyte growth factor (HGF), leptin, PDGFAB/BB, placental growth factor (PlGF), thrombospondin 2, TIMP4, and uPA (Fig. 3C).

Regarding cytokine secretion, adiponectin, CD14, CD31, CD40 ligand (CD154), chitinase 3‐like 1, complement factor D, EMMPRIN (CD147, basigin), GDF‐15, IGFBP‐2, IL1RA, IP‐10, M‐CSF, MIF, MIG (CXCL9), MIP‐3β (CCL19), PDGFAA, RANTES (CCL5), RBP‐4, relaxin‐2, ST2, TNF‐α, and uPAR (CD87) were significantly more abundant in MAT supernatants compared to SVF ones (Fig. 4B). In the cytokine arrays, the increased secretion of endoglin, HGF, leptin, and DPPIV by MAT compared to SVF observed in the angiogenesis array was replicated (Fig. 4B).

## Discussion

AT has long been used in the clinic as a plain, unprocessed tissue graft. Adopting the enzymatically produced adipocyte free SVF was seen as a major improvement, even more so as the AT‐derived SVF can be grown in culture into therapeutically potent MSCs 59. However the use of dissociated AT‐derived SVF, cultured or not into MSCs, for applications in regenerative medicine remains empirical as the exact mode of action of these cells is still obscure, with progenitor potential, trophic, secretory, and immunomodulatory activities being diversely suggested to mediate tissue regeneration and repair.

As a middle path between intact AT and single cell suspensions, mechanical dissociation of AT into microclusters (exemplified by the Lipogems system) has yielded a product of high therapeutic value. In the present study, we used immunohistochemistry to describe MAT clusters and compared those, side by side, with SVF derived from the same donors in terms of perivascular presumptive MSC content and secretory activity in culture. As expected, the microanatomy of MAT is essentially similar to that of intact AT, with capillaries and microvessels organized and distributed between adipocytes, and pericytes wrapped around endothelial cells. However, quantification by flow cytometry of pericytes and adventitial stromal cells revealed that pericytes are encountered at a higher frequency in MAT than in LPA and derived SVF, confirming previous observations 44, 60. Conversely, LPA contains more adventitial stromal cells than MAT preparations do. This suggests that microvessels are spared by microfragmentation, and preserved in MAT, whereas larger arteries and veins, encircled by the *tunica adventitia*, are partially lost after mechanical dissociation. While both purified pericytes and adventitial stromal cells yield MSCs in vitro 13, 18, their respective contributions to long‐term conventional MSC cultures are unknown. However, description of gene expression in single cells has revealed that adventitial stromal cells are developmentally more primitive than pericytes 29, although we and other investigators have previously documented the strong regenerative potential of pericytes purified from AT 20, 24, 61, 62, 63, 64. Therefore, if the therapeutic value of Lipogems is related to pericyte enrichment, those cells might exert their regenerative action more indirectly, via growth factor secretion, than as progenitor cells. This hypothesis was tested by characterizing, qualitatively and quantitatively, growth factors and cytokines produced in culture by MAT preparations and SVFs obtained from the same AT samples. The overall conclusion is that Lipogems‐derived MAT produces higher amounts of many tested growth factors and cytokines, as compared with enzymatically dissociated SVF. As a main difference between MAT and SVF, only the former contains adipocytes; therefore, as expected, MAT but not SVF culture supernatants contained factors secreted by adipocytes: adiponectin, which regulates several metabolic cascades 65; complement factor D 66; RBP‐4, a retinol binding protein 67; the anti‐inflammatory Il‐1RA 68; and leptin, the satiety regulating adipokine 69. No other molecules detected in MAT supernatants are known to be secreted by adipocytes. Interestingly, several of the factors present in higher amounts in MAT supernatants stimulate angiogenesis, hence may indirectly support tissue regeneration: angiogenin 70, endoglin 71, the VEGF family member PlGF 72, the multifunctional HGF 73, and PDGF 74. The insulin‐like growth factor binding protein IGFBP2, which is much more abundant in MAT supernatants, also positively regulates angiogenesis through modulation of VEGF expression 75, and TNFα, whose secretion is upregulated in MAT cultures, can promote new vessel formation 76. These data support and extend the results of a previous study in which Lipogems‐derived MAT culture supernatants were observed to stimulate endothelial cell (HUVEC) proliferation and tube formation in vitro; accordingly, angiopoietin‐1 and ‐2 were detected in higher amounts in these MAT supernatants, as compared to culture supernatants of AT‐derived MSCs 64. In the present experiments, three inhibitors of angiogenesis were also detected, albeit in very small amounts, in MAT supernatants: IP‐10 (CXCL10) 77, angiostatin 78, and thrombospondin 2 79.

Remarkably, higher amounts of diverse mediators of immuno‐inflammation, leukocyte recruitment, and migration were detected in MAT supernatants, as compared to SVF cultures: GDF‐15, a regulator of inflammation and biomarker in diverse pathologies 80; MIF, which controls inflammation and innate immunity 81; MIG (CXCL9), a T‐cell chemoattractant 82; MIP3β (CCL19), involved in immune cell migration 83; RANTES (CCL5), a chemokine chemotactic for multiple leukocytes 84; and CD40 ligand (CD154), which binds to CD40 on antigen presenting cells 85. It is known that adipose tissue can support substantial inflammation 86, hence the presence in culture supernatants of these many players of immune‐inflammatory responses. However, how these factors may influence tissue regeneration, following autologous transplantation, is unknown, and interpretation is complicated by the fact that many of these molecules can play multiple distinct roles. For instance, the CCL5 chemokine, which recruits leukocytes at the site of inflammation, can also promote angiogenesis 87. Chitinase 3‐like‐1, dramatically overexpressed in MAT supernatants, can stimulate blood vessel formation in tumors, besides playing major roles in inflammation, angiogenesis, and tissue remodeling 88.

Why does MAT secrete more growth factors and cytokines than SVF cultured under the same conditions? Besides adipocytes, secretory cells within fat tissue include hematopoietic cells, responsible for the production of most factors involved in the regulation of immuno‐inflammatory reactions, endothelial and perivascular cells, including pericytes, and other cell compartments loosely designated as stromal or “mesenchymal.” The demonstration that mesenchymal stem/stromal cells are of perivascular origin has supported the development of a model whereby pericytes and other perivascular cells can, in adverse pathologic conditions, lose contact with blood vessel walls, migrate away from blood vessels and become reprogrammed into regenerative cells 89, playing this role as tissue progenitors 13, 18, niche cells for lineage specific stem cells 24, “medicinal secretory cells” producing trophic factors 57, as well as scarring pro‐fibrotic cells 35, 36, 37, 38. The present results suggest that AT resident regenerative cells perform these functions much more efficiently when maintained in the intact perivascular environment, such as that provided by the Lipogems mechanical fragmentation system, than following digestion and culture. Notably, it is known that tissue enzymatic dissociation can cause changes in gene expression 90 and exosome content 91 and this research reveals how severely AT residing native MSCs can be affected when reduced to single cell suspensions. Taken together, these data reveal differences in physically fragmented AT, compared to SVF, which provide the foundations on which to build an explanation of the former's therapeutic superiority. Further investigations, not only in culture but also involving experiments in animals, are needed to confirm the effect of enzymatic dissociation on native MSCs, giving more insight into the therapeutic effect of MAT.

## Author Contributions

B.V.: conception and design, collection and assembly of data, data analysis and interpretation, manuscript writing; I.S.: conception and design, collection and assembly of data; H.L.: collection and assembly of data; L.Y.: provision of study material; N.K.: collection and assembly of data; C.T.: conception and design, provision of study material; B.P.: conception and design, data analysis and interpretation, manuscript writing, final approval of manuscript.

## Disclosure Of Potential Conflicts Of Interest

L.Y. declared research funding with Small Pump Priming Grant, Royal College of Surgeons Edinburgh. C.T. is the President and founder of Lipogems and declared employment, patent holder and stock ownership. B.P. declared consultancy and researcher funding. All other authors indicated no potential conflicts of interest.

## References

[1] Strong AL , Cederna PS , Rubin P et al. The current state of fat grafting: A review of harvesting, processing, and injection techniques. Plast Reconstr Surg 2015;136:897–912. 26086386 10.1097/PRS.0000000000001590 PMC4833505

[2] Tremolada C , Palmieri G , Ricordi C . Adipocyte transplantation and stem cells: Plastic surgery meets regenerative medicine. Cell Transplant 2010;19:1217–1223. 20444320 10.3727/096368910X507187

[3] Zuk PA , Zhu M , Mizuno H et al. Multilineage cells from human adipose tissue: Implications for cell‐based therapies. Tissue Eng 2001;7:211–228. 11304456 10.1089/107632701300062859

[4] Schäffler A , Büchler C . Concise review: Adipose tissue‐derived stromal cells—Basic and clinical implications for novel cell‐based therapies. Stem Cells 2007;25:818–827. 17420225 10.1634/stemcells.2006-0589

[5] Yañez R , Lamana ML , García‐Castro J et al. Adipose tissue‐derived mesenchymal stem cells have in vivo immunosuppressive properties applicable for the control of the graft‐versus‐host disease. Stem Cells 2006;24:2582–2591. 16873762 10.1634/stemcells.2006-0228

[6] Dominici M , Le Blanc K , Mueller I et al. Minimal criteria for defining multipotent mesenchymal stromal cells. The International Society for Cellular Therapy position statement. Cytotherapy 2006;8:315–317. 16923606 10.1080/14653240600855905

[7] Gronthos S , Mankani M , Brahim J et al. Postnatal human dental pulp stem cells (DPSCs) in vitro and in vivo. Proc Natl Acad Sci U S A 2000;97:13625–13630. 11087820 10.1073/pnas.240309797 PMC17626

[8] Lama VN , Smith L , Badri L et al. Evidence for tissue‐resident mesenchymal stem cells in human adult lung from studies of transplanted allografts. J Clin Invest 2007;117:989–996. 17347686 10.1172/JCI29713 PMC1810571

[9] Zheng B , Cao B , Crisan M et al. Prospective identification of myogenic endothelial cells in human skeletal muscle. Nat Biotechnol 2007;25:1025–1034. 17767154 10.1038/nbt1334

[10] Gargett CE , Schwab KE , Zillwood RM et al. Isolation and culture of epithelial progenitors and mesenchymal stem cells from human endometrium. Biol Reprod 2009;80:1136–1145. 19228591 10.1095/biolreprod.108.075226 PMC2849811

[11] Shi S , Gronthos S . Perivascular niche of postnatal mesenchymal stem cells in human bone marrow and dental pulp. J Bone Miner Res 2003;18:694–704. 10.1359/jbmr.2003.18.4.696 12674330

[12] Schwab KE , Gargett CE . Co‐expression of two perivascular cell markers isolates mesenchymal stem‐like cells from human endometrium. Hum Reprod 2007;22:2903–2911. 17872908 10.1093/humrep/dem265

[13] Crisan M , Yap S , Casteilla L et al. A perivascular origin for mesenchymal stem cells in multiple human organs. Cell Stem Cell 2008;3:301–313. 18786417 10.1016/j.stem.2008.07.003

[14] da Silva ML , Caplan AI , Nardi NB . In search of the in vivo identity of mesenchymal stem cells. Stem Cells 2008;26:2287–2299. 18566331 10.1634/stemcells.2007-1122

[15] Traktuev DO , Merfeld‐Clauss S , Li J et al. A population of multipotent CD34‐positive adipose stromal cells share pericyte and mesenchymal surface markers, reside in a periendothelial location, and stabilize endothelial networks. Circ Res 2008;102:77–85. 17967785 10.1161/CIRCRESAHA.107.159475

[16] Stefanska A , Kenyon C , Christian HC et al. Human kidney pericytes produce renin. Kidney Int 2016;90:1251–1261. 27678158 10.1016/j.kint.2016.07.035 PMC5126097

[17] Guimarães‐Camboa N , Cattaneo P , Sun Y et al. Pericytes of multiple organs do not behave as mesenchymal stem cells in vivo. Cell Stem Cell 2017;20:345–359. 28111199 10.1016/j.stem.2016.12.006 PMC5337131

[18] Corselli M , Chen CW , Sun B et al. The tunica adventitia of human arteries and veins as a source of mesenchymal stem cells. Stem Cells Dev 2012;21:1299–1308. 21861688 10.1089/scd.2011.0200 PMC3353742

[19] de Souza LEB , de Malta TM , Kashima Haddad S et al. Mesenchymal stem cells and pericytes: To what extent are they related? Stem Cells Dev 2016;25:1843–1852. 27702398 10.1089/scd.2016.0109

[20] James AW , Zara JN , Zhang X et al. Perivascular stem cells: A prospectively purified mesenchymal stem cell population for bone tissue engineering. Stem Cells Translational Medicine 2012;1:510–519. 23197855 10.5966/sctm.2012-0002 PMC3659717

[21] Kramann R , Goettsch C , Wongboonsin J et al. Adventitial MSC‐like cells are progenitors of vascular smooth muscle cells and drive vascular calcification in chronic kidney disease. Cell Stem Cell 2016;19:628–642. 27618218 10.1016/j.stem.2016.08.001 PMC5097006

[22] Hindle P , Khan N , Biant L et al. The infra‐patellar fat pad as a source of perivascular stem cells with increased chondrogenic potential for regenerative medicine. Stem Cells Translational Medicine 2017;6:77–87. 28170170 10.5966/sctm.2016-0040 PMC5442731

[23] Crisan M , Corselli M , Chen WCW et al. Perivascular cells for regenerative medicine. J Cell Mol Med 2012;16:2851–2860. 22882758 10.1111/j.1582-4934.2012.01617.x PMC4393715

[24] Corselli M , Chin C , Parekh C et al. Perivascular support of human hematopoietic stem/progenitor cells. Blood 2013;121:2891–2901. 23412095 10.1182/blood-2012-08-451864 PMC3707421

[25] Zimmerlin L , Donnenberg VS , Rubin JP et al. Mesenchymal markers on human adipose stem/progenitor cells. Cytometry 2013;83:134–140. 23184564 10.1002/cyto.a.22227 PMC4157311

[26] Vezzani B , Pierantozzi E , Sorrentino V . Not all pericytes are born equal: pericytes from human adult tissues present different differentiation properties. Stem Cells Dev 2016;25:1549–1558. 10.1089/scd.2016.0177 27549576

[27] Vezzani B , Pierantozzi E , Sorrentino V . Mesenchymal stem cells: From the perivascular environment to clinical applications. Histol Histopathol 2018;7:11998. 10.14670/HH-11-998 29733091

[28] da Silva ML , Maistro Malta T , Panepucci RA et al. Transcriptomic comparisons between cultured human adipose tissue‐derived pericytes and mesenchymal stromal cells. Genom Data 2016;7:20–25. 26981353 10.1016/j.gdata.2015.11.009 PMC4778596

[29] Hardy WR , Moldovan NI , Moldovan L et al. Transcriptional networks in single perivascular cells sorted from human adipose tissue reveal a hierarchy of mesenchymal stem cells. Stem Cells 2017;35:1273–1289. 28233376 10.1002/stem.2599

[30] Tang W , Zeve D , Suh JM et al. White fat progenitor cells reside in the adipose vasculature. Science 2008;322:583–586. 18801968 10.1126/science.1156232 PMC2597101

[31] Feng J , Mantesso A , De Bari C et al. Dual origin of mesenchymal stem cells contributing to organ growth and repair. Proc Natl Acad Sci U S A 2011;108:6503–6508. 21464310 10.1073/pnas.1015449108 PMC3081015

[32] Zhao H , Feng J , Seidel K et al. Secretion of shh by a neurovascular bundle niche supports mesenchymal stem cell homeostasis in the adult mouse incisor. Cell Stem Cell 2014;14:160–173. 24506883 10.1016/j.stem.2013.12.013 PMC3951379

[33] Dellavalle A , Maroli G , Covarello D et al. Pericytes resident in postnatal skeletal muscle differentiate into muscle fibres and generate satellite cells. Nat Commun 2011;2:499. 21988915 10.1038/ncomms1508

[34] Krautler NJ , Kana V , Kranich J et al. Follicular dendritic cells emerge from ubiquitous perivascular precursors. Cell 2012;150:194–206. 22770220 10.1016/j.cell.2012.05.032 PMC3704230

[35] Göritz C , Dias DO , Tomilin N et al. A pericyte origin of spinal cord scar tissue. Science 2011;333:238–242. 21737741 10.1126/science.1203165

[36] Dulauroy S , Di Carlo SE , Langa F et al. Lineage tracing and genetic ablation of ADAM12(+) perivascular cells identify a major source of profibrotic cells during acute tissue injury. Nat Med 2012;18:1262–1270. 22842476 10.1038/nm.2848

[37] Kramann R , Schneider RK , DiRocco DP et al. Perivascular Gli1+ progenitors are key contributors to injury‐induced organ fibrosis. Cell Stem Cell 2015;16:51–66. 25465115 10.1016/j.stem.2014.11.004 PMC4289444

[38] Murray IR , Gonzalez ZN , Baily J et al. αv integrins on mesenchymal cells critically regulate skeletal and cardiac muscle fibrosis. Nat Commun 2017;8:1118. 29061963 10.1038/s41467-017-01097-z PMC5653645

[39] Muraglia A , Cancedda R , Quarto R . Clonal mesenchymal progenitors from human bone marrow differentiate in vitro according to a hierarchical model. J Cell Sci 2000;113:1161–1166. 10704367 10.1242/jcs.113.7.1161

[40] Guilak F , Lott KE , Awad HA et al. Clonal analysis of the differentiation potential of human adipose‐derived adult stem cells. J Cell Physiol 2006;206:229–237. 16021633 10.1002/jcp.20463

[41] Russell KC , Phinney DG , Lacey MR et al. In vitro high‐capacity assay to quantify the clonal heterogeneity in trilineage potential of mesenchymal stem cells reveals a complex hierarchy of lineage commitment. Stem Cells 2010;28:788–798. 20127798 10.1002/stem.312

[42] Manini I , Gulino L , Gava B et al. Multi‐potent progenitors in freshly isolated and cultured human mesenchymal stem cells: A comparison between adipose and dermal tissue. Cell Tissue Res 2011;344:85–95. 21336533 10.1007/s00441-011-1139-0

[43] Tabit CJ , Slack GC , Fan K et al. Fat grafting versus adipose‐derived stem cell therapy: Distinguishing indications, techniques, and outcomes. Aesthetic Plast Surg 2012;36:704–713. 22069062 10.1007/s00266-011-9835-4

[44] Bianchi F , Maioli M , Leonardi E et al. A new non enzymatic method and device to obtain a fat tissue derivative highly enriched in pericyte‐like elements by mild mechanical forces from human lipoaspirates. Cell Transplant 2013;2:2063–2077. 10.3727/096368912X657855 23051701

[45] Raffaini M , Tremolada C . Micro fractured and purified adipose tissue graft (Lipogems®) can improve the orthognathic surgery outcomes both aesthetically and in postoperative healing. CellR4 2014;2:e1118.

[46] Cestaro G , De Rosa M , Massa S et al. Intersphincteric anal lipofilling with micro‐fragmented fat tissue for the treatment of faecal incontinence: preliminary results of three patients. Wideochir Inne Tech Maloinwazyjne 2015;10:337–341. 26240640 10.5114/wiitm.2014.47435 PMC4520831

[47] Fantasia J , Chen H , Santos Cortes JA . Microfractured and purified adipose tissue (Lipogems™ system) injections for treatment of atrophic vaginitis. J Urol Res 2016;3:1073–1075.

[48] Saibene AM , Pipolo C , Lorusso R et al. Transnasal endoscopic microfractured fat injection in glottic insufficiency. B‐ENT 2015;11:229–234. 26601557

[49] Giori A , Tremolada C , Vailati R et al. Recovery of function in anal incontinence after micro‐fragmented fat graft (Lipogems®) injection: Two years follow up of the first 5 cases. CellR4 2015;3:e1544.

[50] Tremolada C , Beltrami G , Magri A et al. Adipose mesenchymal stem cells and regenerative adipose tissue graft (Lipogems®) for musculoskeletal regeneration. Eur J Muscoloskeletal Dis 2014;3:57–67.

[51] Striano RD , Chen H , Bilbool N et al. Non‐responsive knee pain with osteoarthritis and concurrent meniscal disease treated with autologous micro‐fragmented adipose tissue under continuous ultrasound guidance. CellR4 2015;3:e1690.

[52] Randelli P , Menon A , Ragone V et al. Lipogems product treatment increases the proliferation rate of human tendon stem cells without affecting their stemness and differentiation capability. Stem Cells Int 2016;2016:4373410. 27057170 10.1155/2016/4373410 PMC4736573

[53] Bianchi F , Olivi E , Baldassarre M et al. Lipogems®, a new modality off at tissue handling to enhance tissue repair in chronic hind limb ischemia. CellR4 2014;2:e1289.

[54] Benzi R , Marfia G , Bosetti M et al. Microfractured lipoaspirate may help oral bone and soft tissue regeneration: A case report. CellR4 2015;3:e1583.

[55] Schindelin J , Arganda‐Carreras I , Frise E et al. Fiji: An open‐source platform for biological image analysis. Nat Methods 2012;9:676–682. 22743772 10.1038/nmeth.2019 PMC3855844

[56] West CC , Hardy WR , Murray IR et al. Prospective isolation of perivascular stem cells (PSC) from human adipose tissue: Cell population metrics across a large cohort of diverse demographics. Stem Cell Res Ther 2016;7:47. 27029948 10.1186/s13287-016-0302-7 PMC4815276

[57] Caplan AI . Mesenchymal stem cells: Time to change the name! Stem Cells Translational Medicine 2017;6:1445–1451. 28452204 10.1002/sctm.17-0051 PMC5689741

[58] Phinney DG , Pittenger MF . Concise review: MSC‐derived exosomes for cell‐free therapy. Stem Cells 2017;35:851–858. 28294454 10.1002/stem.2575

[59] Zimmerlin L , Donnenberg VS , Pfeifer ME et al. Stromal vascular progenitors in adult human adipose tissue. Cytometry A 2010;77:22–30. 19852056 10.1002/cyto.a.20813 PMC4148047

[60] Ceserani V , Ferri A , Berenzi A et al. Angiogenic and anti‐inflammatory properties of micro‐fragmented fat tissue and its derived mesenchymal stromal cells. Vasc Cell 2016;18:8–3. 10.1186/s13221-016-0037-3 PMC4991117 27547374

[61] Mendel TA , Clabough EBD , Kao DS et al. Pericytes derived from adipose‐derived stem cells protect against retinal vasculopathy. PLoS One 2013;8:e691. 10.1371/journal.pone.0065691 PMC3669216 23741506

[62] König MA , Canepa DD , Cadosch D et al. Direct transplantation of native pericytes from adipose tissue: A new perspective to stimulate healing in critical size bone defects. Cytotherapy 2016;18:41–52. 26563474 10.1016/j.jcyt.2015.10.002

[63] West CC , Hardy WR , Murray IR et al. Prospective purification of perivascular presumptive mesenchymal stem cells from human adipose tissue: Process optimization and cell population metrics across a large cohort of diverse demographics. Stem Cell Res Ther 2016;7:47. 27029948 10.1186/s13287-016-0302-7 PMC4815276

[64] Zhang J , Du C , Guo W et al. Adipose tissue‐derived pericytes for cartilage tissue engineering. Curr Stem Cell Res Ther 2017;12:513–521. 28325151 10.2174/1574888X12666170321111211

[65] Nigro E , Scudiero O , Monaco ML et al. New insight into adiponectin role in obesity and obesity‐related diseases. Biomed Res Int 2014;2014:658913. 25110685 10.1155/2014/658913 PMC4109424

[66] Ronti T , Lupattelli G , Mannarino E . The endocrine function of adipose tissue: An update. Clin Endocrinol (Oxf) 2006;64:355–365. 16584505 10.1111/j.1365-2265.2006.02474.x

[67] Yang Q , Graham TE , Mody N et al. Serum retinol binding protein 4 contributes to insulin resistance in obesity and type 2 diabetes. Nature 2005;436:356–362. 16034410 10.1038/nature03711

[68] Arend WP . The balance between IL‐1 and IL‐1Ra in disease. Cytokine Growth Factor Rev 2003;13:323–340. 10.1016/s1359-6101(02)00020-5 12220547

[69] Fasshauer M , Blüher M . Adipokines in health and disease. Trends Pharmacol Sci 2015;36:461–470. 26022934 10.1016/j.tips.2015.04.014

[70] Sheng J , Xu Z . Three decades of research on angiogenin: A review and perspective. Acta Biochim Biophys Sin 2016;48:399–410. 26705141 10.1093/abbs/gmv131 PMC4888354

[71] Nassiri F , Cusimano MD , Scheithauer BW et al. Endoglin (CD105): A review of its role in angiogenesis and tumor diagnosis, progression and therapy. Anticancer Res 2011;31:2283–2290. 21737653

[72] De Falco S , Gigante B , Persico GM . Structure and function of placental growth factor. Trends Cardiovasc Med 2002;12:241–246. 12242046 10.1016/s1050-1738(02)00168-8

[73] Bussolino F , Di Renzo MF , Ziche M et al. Hepatocyte growth factor is a potent angiogenic factor which stimulates endothelial cell motility and growth. J Cell Biol 1992;119:629–641. 1383237 10.1083/jcb.119.3.629 PMC2289675

[74] Liu ZJ , Battinelli E , Sparger KA et al. Novel insights into the role of platelet angiogenic growth factors on the regulation of normal vascular development. Paper presented at: ASH 59th Annual Meeting and Exposition, Atalanta, 2017.

[75] Bruns AF , Smith J , Yuldasheva N et al. Insulin‐like growth factor binding protein 2 (igfbp2): A positive regulator of angiogenesis? BMJ Heart 2017;103:A121.

[76] Bodnar RJ . Chemokine regulation of angiogenesis during wound healing. Adv Wound Care 2015;4:641–650. 10.1089/wound.2014.0594 PMC4620517 26543678

[77] Yates‐Binder CC , Rodgers M , Jaynes J et al. An IP‐10 (CXCL10)‐derived peptide inhibits angiogenesis. PLoS One 2012;7:e40812. 22815829 10.1371/journal.pone.0040812 PMC3397949

[78] Cao Y , Ji RW , Davidson D et al. Kringle domains of human angiostatin. Characterization of the anti‐proliferative activity on endothelial cells. J Biol Chem 1996;271:29461–29467. 8910613 10.1074/jbc.271.46.29461

[79] Volpert OV , Tolsma SS , Pellerin S et al. Inhibition of angiogenesis by thrombospondin‐2. Biochem Biophys Res Commun 1995;217:326–332. 8526929 10.1006/bbrc.1995.2780

[80] O’Rahilly S . GDF15—From biomarker to allostatic hormone. Cell Metab 2017;26:807–808. 29195860 10.1016/j.cmet.2017.10.017

[81] Calandra T , Roger T . Macrophage migration inhibitor factor: A regulator of innate immunity. Nat Rev Immunol 2003;3:791–800. 14502271 10.1038/nri1200 PMC7097468

[82] Müller M , Carter S , Hofer MJ et al. The chemokine receptor CXCR3 and its ligands CXCL9, CXCL10 and CXCL11 in neuroimmunity—A tale of conflict and conundrum. Neuropathol Appl Neurobiol 2010;36:368–387. 20487305 10.1111/j.1365-2990.2010.01089.x

[83] Comerford I , Harata‐Lee Y , Bunting MD et al. A myriad of functions and complex regulation of the CCR7/CCL19/CCL21 chemokine axis in the adaptive immune system. Cytokine Growth Factor Rev 2013;24:269–283. 23587803 10.1016/j.cytogfr.2013.03.001

[84] Appay V , Rowland‐Jones SL . RANTES: A versatile and controversial chemokine. Trends Immunol 2001;22:83–87. 11286708 10.1016/s1471-4906(00)01812-3

[85] Schonbeck U , Libby P . The CD40/CD154 receptor/ligand dyad. Cell Mol Life Sci 2001;58:4–43. 11229815 10.1007/PL00000776 PMC11146501

[86] Crewe C , An YA , Scherer PE . The ominous triad of adipose tissue dysfunction: Inflammation, fibrosis, and impaired angiogenesis. J Clin Invest 2017;127:74–82. 28045400 10.1172/JCI88883 PMC5199684

[87] Suffee N , Richard B , Hlawaty H et al. Angiogenic properties of the chemokine RANTES/CCL5. Biochem Soc Trans 2011;39:1649–1653. 22103502 10.1042/BST20110651

[88] Francescone R , Ngernyuang N , Yan W et al. Tumor‐derived mural‐like cells coordinate with endothelial cells: role of YKL‐40 in mural cell‐mediated angiogenesis. Oncogene 2014;33:2110–2122. 23665676 10.1038/onc.2013.160 PMC3926897

[89] Murray IR , Peault B . Q&A: What is a mesenchymal stem cell, and why is it important? BMC Biol 2015;13:99. 26596888 10.1186/s12915-015-0212-7 PMC4656175

[90] Van den Brink SC , Sage F , Vértesy Á et al. Single‐cell sequencing reveals dissociation‐induced gene expression in tissue subpopulations. Nat Methods 2017;14:935–936. 28960196 10.1038/nmeth.4437

[91] García‐Contreras M , Messaggio F , Jimenez O et al. Differences in exosome content of human adipose tissue processed by non‐enzymatic and enzymatic methods. CellR4 2014;3:e1423.

